# Psychosocial Outcomes of Microwave Thermolysis for Axillary Hyperhidrosis and Bromhidrosis in Adolescents: A Retrospective Study

**DOI:** 10.1111/jocd.71114

**Published:** 2026-08-03

**Authors:** Sheng‐Ni Chen, Sindy Hu, Chun‐Yu Cheng, Shyue‐Luen Chang, Chau‐Yee Ng, Ya‐Ching Chang, Min‐Hui Chi, Jennifer Wu, Sze‐Wen Ting, Fang‐Ying Wang, Yau‐Li Huang

**Affiliations:** ^1^ Department of Dermatology Chang Gung Memorial Hospital Linkou, Taipei, Taoyuan, Keelung Taiwan; ^2^ College of Medicine Chang Gung University Taoyuan Taiwan; ^3^ Department of Aesthetic Medicine Chang Gung Memorial Hospital Taoyuan Taiwan; ^4^ Department of Aesthetic Medicine Chang Gung Clinic Taipei Taiwan; ^5^ Department of Dermatology Xiamen Chang Gung Memorial Hospital Xiamen China; ^6^ Department of Dermatology and Aesthetic Medicine Center Jen‐Ai Hospital Taichung Taiwan; ^7^ Department of Cosmetic Science Chang Gung University of Science and Technology Taoyuan Taiwan

**Keywords:** adolescents, axillary hyperhidrosis, bromhidrosis, microwave thermolysis, psychosocial impact, quality of life

## Abstract

**Background:**

Primary axillary hyperhidrosis and bromhidrosis in adolescents cause significant physical discomfort and psychosocial distress, negatively impacting emotional well‐being and social function. While microwave thermolysis is effective in adults, its psychological benefits in adolescents remain understudied.

**Aims:**

To evaluate the clinical efficacy of microwave thermolysis in adolescents and investigate its impact on quality of life, anxiety, and depression.

**Patients/Methods:**

This retrospective study included 21 adolescents (mean age 15.21 years) treated with microwave thermolysis. Clinical severity was assessed using the Hyperhidrosis Disease Severity Scale (HDSS) and a bromhidrosis grading scale. Psychosocial outcomes were evaluated using the Dermatology Life Quality Index (DLQI), a modified Quality of Life (QoL) questionnaire, and the Hospital Anxiety and Depression Scale (HADS) at baseline and 1 month post‐treatment.

**Results:**

The treatment yielded significant clinical improvement, with HDSS scores decreasing from 3.19 to 1.33 (*p* < 0.0001) and bromhidrosis severity dropping from 4.19 to 0.48 (*p* < 0.0001). Psychosocial burden was markedly reduced: DLQI scores improved from 17.38 to 10.71 (*p* < 0.0001), and QoL scores increased from 52.10 to 84.95 (*p* = 0.027). Significant reductions were observed in HADS‐anxiety (14.05 to 1.67, *p* < 0.0001) and HADS‐depression scores (7.48 to 1.05, *p* < 0.0001). All cases reported no major side effects.

**Conclusions:**

Microwave thermolysis is a highly effective treatment for adolescent axillary hyperhidrosis and bromhidrosis. Beyond symptom relief, it significantly alleviates anxiety and depression while enhancing quality of life and social function. These findings support its use as a primary therapeutic option for this vulnerable population.

## 1 Introduction

Primary focal hyperhidrosis is a chronic autonomic disorder diagnosed based on well‐established clinical criteria. It is characterized by focal, visible, and excessive sweating of at least 6 months' duration without an identifiable secondary cause [1]. According to standard diagnostic guidelines, the condition typically presents with bilateral and relatively symmetrical involvement and occurs with a frequency of at least one episode per week. Crucially, the disease often manifests early in life, with an age of onset usually under 25 years, making it a prevalent concern in the pediatric and adolescent population. Other key features supporting the diagnosis include the impairment of daily activities, a positive family history, and the cessation of focal sweating during sleep [1, 2].

Beyond physical discomfort, primary axillary hyperhidrosis (PAH) and its frequently associated condition, bromhidrosis, impose a substantial psychosocial burden on children and adolescents, profoundly affecting emotional well‐being, interpersonal relationships, and daily functioning [3, 4]. The prevalence in the United States has been reported as 1.4%; however, 20% to 33% of individuals with AH indicate that their daily activities are impacted [4]. Severe PAH, often defined as a score of 3 or 4 on the Hyperhidrosis Disease Severity Scale (HDSS), can be particularly debilitating. Multiple studies have demonstrated that hyperhidrosis is frequently linked to psychological and functional impairments, including anxiety, sleep disruption, daytime fatigue, and depressive symptoms [5, 6]. Among school‐aged individuals, excessive sweating may hinder everyday academic tasks, particularly handwriting and other fine motor activities. The social consequences are equally significant; patients often report distress during interpersonal interactions and socializing with peers [3, 7, 8]. Furthermore, the condition exerts a heavy emotional toll, leading to reduced self‐confidence and social avoidance [7]. Adolescents may constantly worry that others will notice their sweating or odor, causing them to withdraw from social activities to prevent embarrassment.

Regarding management, conventional treatments have included systemic agents such as oxybutynin and clonidine, as well as botulinum toxin A (BTX‐A) injections. Various surgical techniques, ranging from the local excision of apocrine and eccrine glands to sympathectomy, have also been employed. However, the limitations of these approaches, including the temporary nature of pharmacological results and the invasiveness and pain associated with surgery, often limit patient acceptance and willingness to undergo treatment. Recently, microwave thermolysis has emerged as an effective non‐invasive modality [5, 9, 10, 11]. By delivering microwave energy to thermally destroy axillary eccrine and apocrine glands, this treatment achieves a long‐term reduction in both sweating and malodor. While studies have demonstrated that this treatment significantly improves quality of life, anxiety, and depression symptoms in adult patients with severe PAH, its specific impact on the adolescent population remains understudied [7, 12]. Notably, microwave thermolysis is not currently FDA‐cleared for patients younger than 18 years. Although clinical cases suggest symptom improvement in minors, there is a lack of systematic evidence evaluating the psychological and social functional changes in this specific age group.

Therefore, the aim of this study was to investigate patient‐reported outcome measures (PROMs) in adolescents with axillary hyperhidrosis and bromhidrosis undergoing microwave thermolysis. We sought to validate the clinical efficacy of this treatment in reducing symptoms and to comprehensively evaluate its impact on psychological health—including anxiety and depression—as well as social function. These findings aim to provide scientific evidence to support the expanded indication of microwave thermolysis for the adolescent population.

## 2 Materials and Methods

### 2.1 Data Sources and Study Participants

A retrospective review of patients treated with microwave thermolysis at the Department of Aesthetic Medicine, Chang Gung Memorial Hospital, between January 2024 and December 2024 was conducted. Eligibility criteria for this study included: (1) age between 12 and 17 years at the time of treatment; (2) clinical evidence of physical maturation, including the presence of axillary hair in all participants and the onset of menarche in all female participants; (3) a diagnosis of primary axillary hyperhidrosis, bromhidrosis, or both; (4) verification of written informed consent signed by parents or legal guardians prior to the procedure; (5) availability of complete medical records, including baseline and 1‐month post‐treatment evaluations of clinical severity and psychosocial status. The selection process focused on developmentally mature adolescents to ensure physiological readiness for the procedure. Patients who had not yet reached these developmental milestones were not candidates for treatment in this cohort. The study protocol was approved by the Institutional Review Board of the Chang Gung Medical Foundation (IRB No. 202502152B0).

### 2.2 Treatment Protocol

Transcutaneous microwave thermolysis was performed using the miraDry system (Miramar Labs, Santa Clara, CA, USA). All procedures were carried out by certified physicians. Local anesthesia was achieved using a standard tumescent solution containing lidocaine, epinephrine, and sodium bicarbonate. Following anesthesia, the treatment area was marked using the system's sizing template. Microwave energy was then delivered to the target area [11, 13]. The template sizes were selected based on each patient's axillary hair‐bearing area, with dimensions ranging from 100 × 50 mm to 150 × 80 mm across the cohort. Each session consisted of a single treatment with two passes of microwave energy delivery per axilla. Unless the patient reported intolerance, the maximum energy setting (Level 5) was utilized to ensure optimal efficacy.

### 2.3 Measures and Outcomes

Outcomes were assessed at baseline (Visit 1) and 1 month post‐treatment (Visit 2). Clinical efficacy was evaluated using the Hyperhidrosis Disease Severity Scale (HDSS) and a specific bromhidrosis severity grading system. The HDSS is a subjective scale ranging from 1 (mild) to 4 (severe), measuring the tolerability of sweating and its interference with daily activities [4]. Bromhidrosis severity was classified on a 4‐point scale tailored for this study: Level 0 indicated no detectable malodor; Level 1 (mild) involved pungent odor detectable only by the patient after exertion; Level 2 (moderate) consisted of strong odor detectable by others at close range during daily activities; and Level 3 (severe) represented strong, pungent odor detectable from a distance even at rest.

To evaluate the psychosocial impact, patients completed three questionnaires. The Dermatology Life Quality Index (DLQI) ranges from 0 to 30, with higher scores indicating greater impairment [14, 15]. Quality of life was further assessed using a modified version of the de Campos Hyperhidrosis Questionnaire, which evaluates functional, social, and emotional domains; total scores range from 20 to 100, with higher scores reflecting better quality of life [16]. Finally, the Hospital Anxiety and Depression Scale (HADS) was used to screen for psychological distress, comprising subscales for anxiety and depression (range 0–21 each), with higher scores indicating higher levels of distress [17, 18].

### 2.4 Statistical Analyses

Statistical analyses and figure generation were performed using GraphPad Prism version 10 (GraphPad Software, Boston, MA, USA). Baseline demographic and clinical characteristics were summarized using descriptive statistics. To evaluate treatment efficacy, changes in clinical severity scores (HDSS and bromhidrosis grading) and PROM scores (DLQI, QoL, and HADS) from baseline to the 1‐month follow‐up were analyzed using paired *t*‐tests. All statistical tests were two‐tailed, with statistical significance defined as a *p*‐value of less than 0.05.

## 3 Results

### 3.1 Demographic and Baseline Characteristics

A total of 21 adolescent patients were included in the study, with a mean age of 15.21 ± 2.09 years. The cohort consisted of 12 females (57.1%) and 9 males (42.9%), and the mean body mass index (BMI) was 20.67 ± 3.71 kg/m^2^. Regarding symptom onset, the majority of patients developed symptoms during secondary education: 33.3% during junior high school (12–15 years) and 57.1% during senior high school (15–18 years). Only 9.6% reported onset during primary school, and no patients experienced symptoms before the age of 6 years. A positive family history of axillary osmidrosis and the presence of wet cerumen were both highly prevalent, each observed in 85.7% of the cohort. Detailed demographic and clinical characteristics are summarized in Table 1.

**TABLE 1:** Demographic and clinical characteristics of included patients.

Patient characteristics	Value (*n* = 21)
Age, mean ± SD (years)	15.21 ± 2.09
Sex, *n* (%)	
Female	12 (57.1%)
Male	9 (42.9%)
BMI (kg/m^2^), mean ± SD	20.67 ± 3.71
Age of onset, *n* (%)	
Preschool (< 6 years)	0
Primary school (6–12 years)	2 (9.6%)
Middle school (12–15 years)	7 (33.3%)
High school (15–18 years)	12 (57.1%)
Family history of axillary osmidrosis, *n* (%)	18 (85.7%)
Wet cerumen, *n* (%)	18 (85.7%)
Baseline HDSS (1–4)	3.19 ± 0.68
Baseline severity of bromhidrosis (score 0–5)	4.19 ± 0.68
Baseline DLQI (4–44)	27.38 ± 5.59
Baseline QoL (20–100)	52.10 ± 13.37
Baseline HADS (0–42)	21.52 ± 9.30

Abbreviations: BMI, body mass index; DLQI, Dermatology Life Quality Index; HADS, Hospital Anxiety and Depression Scale; HDSS, Hyperhidrosis Disease Severity Scale; QoL, quality of life.

### 3.2 Clinical Efficacy and Adverse Events

**FIGURE 1:**
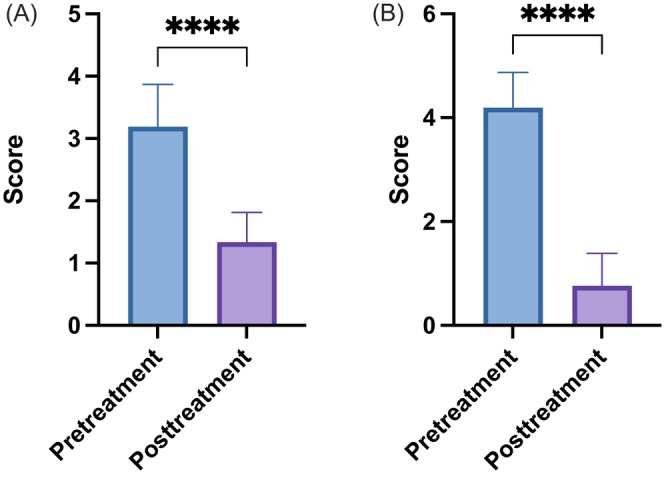
Comparison of clinical disease severity in the adolescent cohort (*N* = 21) before and 1 month after microwave thermolysis. (A) Hyperhidrosis Disease Severity Scale (HDSS) scores. (B) Bromhidrosis severity grading. Both measures demonstrated a statistically significant reduction 1 month after treatment (****p < 0.0001).

Microwave thermolysis yielded significant improvements in clinical symptoms (Table 2). At 1 month post‐treatment, the mean HDSS score decreased significantly from 3.19 ± 0.68 to 1.33 ± 0.76 post‐treatment (*p* < 0.0001). Similarly, the severity of bromhidrosis showed a marked reduction, dropping from 4.19 ± 0.68 to 0.48 ± 0.62 (*p* < 0.0001). Figure 1 illustrates these rapid and sustained reductions in both sweating and malodor severity following the procedure. Follow‐up evaluations of all 21 patients revealed that while mild localized swelling occurred within the initial 1 to 2 weeks post‐procedure, most participants achieved full recovery by the 1‐month mark. Importantly, no serious complications or major adverse effects were observed during the study period. Regarding the safety profile, localized painful swelling at 1 week post‐procedure was reported in 5 cases (23.8%). Ecchymosis and erythema were noted in 9 patients (42.9%), both of which progressively resolved within the 1‐month follow‐up period. All documented adverse events were transient and self‐limiting; no severe complications, such as deep tissue thermal injury or secondary infections, were observed during the study period.

**TABLE 2:** Changes in clinical severity and patient‐reported outcomes following microwave thermolysis.

Outcome measure	Pre‐treatment (mean ± SD)	Post‐treatment (mean ± SD)	*p*
Clinical severity			
HDSS	3.19 ± 0.68	1.33 ± 0.76	< 0.0001
Bromhidrosis severity	4.19 ± 0.68	0.48 ± 0.62	< 0.0001
Psychosocial outcomes			
DLQI	17.38 ± 5.59	10.71 ± 1.90	< 0.0001
QoL	52.10 ± 13.37	84.95 ± 12.31	0.027
HADS			
Total score	21.52 ± 9.30	2.71 ± 4.63	< 0.0001
HADS‐anxiety	14.05 ± 5.46	1.67 ± 2.85	< 0.0001
HADS‐depression	7.48 ± 4.75	1.05 ± 1.86	< 0.0001

*Note:* Data are presented as mean ± SD. *p*‐values were calculated using the paired *t*‐test.

### 3.3 Psychosocial and Quality of Life Outcomes

**FIGURE 2:**
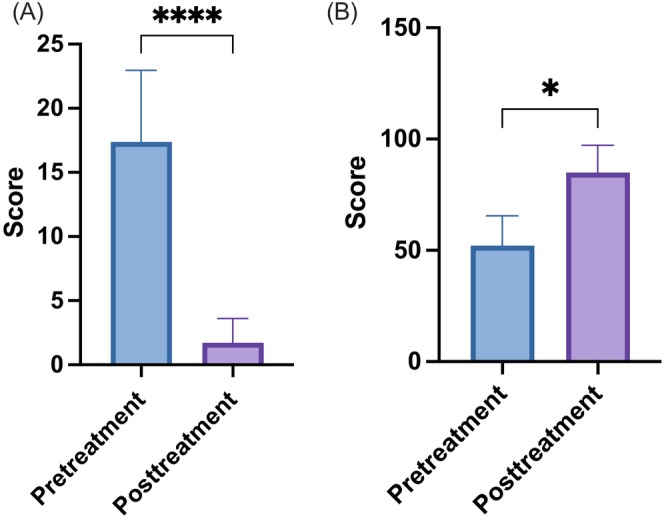
Assessment of quality of life outcomes before and 1 month after microwave thermolysis. (A) Dermatology Life Quality Index (DLQI) scores. (B) Modified Hyperhidrosis quality of life (QoL) questionnaire scores. Both indices showed significant improvement 1 month following therapy (****p < 0.0001 for DLQI and *p = 0.027 for QoL).

**FIGURE 3:**
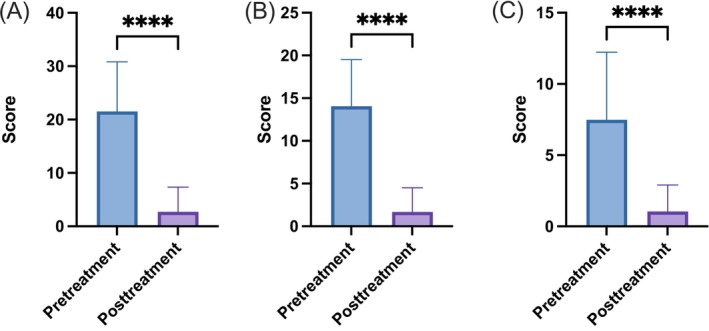
Evaluation of psychological impact using the Hospital Anxiety and Depression Scale (HADS) before and 1 month after treatment. (A) The anxiety subscale scores. (B) The depression subscale scores. (C) The total HADS scores. Significant reductions were observed in all three parameters at the 1‐month post‐treatment evaluation (all ****p < 0.0001).

The treatment also resulted in substantial improvements in patient‐reported psychosocial functioning (Table 2). The mean DLQI score decreased from 17.38 ± 5.59 at baseline to 10.71 ± 1.90 post‐treatment (*p* < 0.0001), indicating a significant reduction in the impact of the disease on daily life. Consistent with this finding, quality of life (QoL) scores significantly improved from 52.10 ± 13.37 to 84.95 ± 12.31 (*p* = 0.027) (Figure 2). Furthermore, psychological distress was markedly alleviated across all domains of the HADS. The total HADS score dropped from 21.52 ± 9.30 to 2.71 ± 4.63 (*p* < 0.0001). Sub‐analysis revealed significant reductions in both the anxiety subscale (from 14.05 ± 5.46 to 1.67 ± 2.85, *p* < 0.0001) and the depression subscale (from 7.48 ± 4.75 to 1.05 ± 1.86, *p* < 0.0001). These trends, depicting the alleviation of anxiety and depressive symptoms, are presented in Figure 3.

## 4 Discussion

To the best of our knowledge, this is the first study to investigate both the clinical efficacy and the psychosocial impact of microwave thermolysis specifically in an adolescent cohort suffering from axillary hyperhidrosis and bromhidrosis. Our findings demonstrate that this non‐invasive modality yielded robust and statistically significant improvements in clinical symptoms. More importantly, these physical improvements translated into profound benefits in quality of life and psychological well‐being, evidenced by marked reductions in anxiety and depression scores.

Historically, conventional management has been challenging. Systemic anticholinergics, such as oxybutynin, are associated with bothersome side effects, including dry mouth, urinary retention, and the risk of glaucoma, often leading to poor adherence [9, 19, 20]. Similarly, while botulinum toxin A (BTX‐A) injections are effective, they provide only temporary relief, require repeated painful administration, and incur high long‐term costs [21]. Surgical options present even greater hurdles: local excision of glands involves a difficult recovery and postoperative pain, while endoscopic thoracic sympathectomy (ETS) is an aggressive procedure with a well‐documented risk of irreversible compensatory hyperhidrosis [22]. These drawbacks often deter patients from seeking or continuing treatment, thereby perpetuating their suffering.

In contrast, microwave thermolysis represents a significant therapeutic advancement. It requires only local tumescent anesthesia, involves minimal invasiveness, and typically achieves substantial symptom reduction after treatment [23, 24]. This efficacy is well‐supported by long‐term data: Micu et al. reported that microwave therapy is a promising long‐term efficient treatment for PAH, demonstrating statistically significant improvement in both HDSS and Hyperhidrosis Quality of Life over 3 years of follow‐up [10]. Furthermore, Grove et al.'s randomized controlled trial revealed that while BTX‐A and microwave thermolysis had similar efficacy on sweat reduction at 1‐year follow‐up, odor reduction, hair reduction, and patient preference were demonstrably superior and sustaining for microwave thermolysis [25]. Tan et al. also documented high success, reporting that 90% of patients met the treatment endpoint of decreasing symptoms to below grade 2 on the HDSS score and achieving at least 50% reduction in odor; supporting evidence from the iodine starch test showed a 99% reduction in the treated region from baseline to 12‐month follow‐up [24]. Our findings in the adolescent cohort align with these established adult benchmarks. The clinical improvements and recovery profiles were comparable between the two groups. Importantly, the recovery process in our 21 adolescent patients was consistent with that observed in adults, and no severe complications were identified during the follow‐up period. While adult literature has documented rare but major complications such as deep axillary burns, nerve injuries, or severe sequelae like necrotizing fasciitis, particularly in patients with lower body mass index, our study observed none of these events [23, 26, 27, 28]. In our cohort, immediate post‐treatment side effects were limited to mild, transient ecchymosis and erythema in 9 patients (42.9%), and localized painful swelling at 1 week in 5 cases (23.8%). All of these minor adverse events were entirely self‐limiting and resolved consistently within the first few weeks without any long‐term sequelae. This high safety profile, combined with the significant psychosocial improvements recorded, suggests that microwave thermolysis is not only efficient but also a generally safe and well tolerated primary therapeutic option for patients under 18 years of age.

The psychosocial implications of treating hyperhidrosis and bromhidrosis during adolescence are profound. These conditions are known to severely impair quality of life, often leading to sleep deprivation, depression, and low self‐esteem [8, 12, 29]. Adolescents represent a particularly vulnerable population because visible sweating or malodor in school environments can trigger intense anxiety and feelings of inferiority. Such chronic distress can negatively impact academic performance and, in severe cases, lead to peer victimization or bullying, which eventually results in social isolation and depressive disorders [8, 29]. Therefore, early and effective intervention is essential to restore a healthy psychosocial developmental trajectory. Although microwave thermolysis is currently not FDA‐cleared for patients under 18 years of age and was performed off‐label in this study, our results indicate that it is a viable and valuable treatment modality for minors. To ensure the highest standards of clinical safety and ethical integrity, every patient in this cohort underwent a rigorous and comprehensive evaluation process. This included in‐depth consultations with both the patients and their parents to ensure a shared understanding of the treatment goals and its off‐label status. In several instances, patients were referred to our clinic by school counselors who had identified significant social impairment and psychosocial distress in these students. Furthermore, formal written informed consent was meticulously obtained from both the adolescents and their legal guardians prior to the procedure. This thorough screening and multidisciplinary communication ensured that the intervention was pursued only when clinically and socially justified. Our findings demonstrate that when implemented with such careful oversight, microwave thermolysis offers significant improvements in both physical symptoms and mental health outcomes without any major adverse events.

Several limitations of this study must be acknowledged. First, the study design was retrospective and conducted at a single center with a relatively small sample size (*n* = 21), which may limit the generalizability of the findings. Second, the assessment of quality of life utilized a modified version of the de Campos questionnaire. As this instrument was originally designed primarily for palmar hyperhidrosis, items related to hand function, such as writing, using touch screens may not fully capture the burden of isolated axillary hyperhidrosis. Consequently, patients with pure axillary symptoms might score favorably in functional domains, potentially leading to an underestimation of the overall disease severity. However, the domains assessing social interaction and emotional impact remain highly relevant and sensitive for this population. Third, the use of patient‐reported outcome measures (PROMs) in a retrospective context may introduce recall bias. Additionally, this study relied exclusively on subjective clinical scales and patient‐reported questionnaires without objective measurements, such as Minor's starch‐iodine testing or gravimetric sweat analysis, which could not be retroactively performed due to the retrospective chart review design. Finally, the major limitation of this study is the brief follow‐up period, which was restricted to 1 month post‐treatment. As skin appendage functions can recover over time, early recurrence of bromhidrosis or hyperhidrosis may occur within 3 to 6 months post‐procedure, presenting an unsatisfactory outcome for adolescent patients and their families. Because long‐term data could not be retroactively retrieved within this cohort due to loss of follow‐up, the sustained long‐term efficacy and true recurrence rate of microwave thermolysis in minors remain to be fully elucidated. Consequently, future prospective, multi‐center trials with extended follow‐up periods (at least 6 to 12 months) are strictly warranted to validate the permanence of both clinical outcomes and psychosocial benefits in this vulnerable population.

In conclusion, this study provides the first evidence supporting microwave thermolysis as an effective and safe modality for adolescents with axillary hyperhidrosis and bromhidrosis. Beyond clinical symptom relief, the treatment yielded profound psychosocial improvements, including enhanced quality of life and reduced anxiety and depression. Considering the significant impact of these conditions on adolescent development, microwave thermolysis offers a comprehensive therapeutic solution targeting both physical severity and emotional well‐being.

## Author Contributions

Sheng‐Ni Chen: conceptualization, methodology, formal analysis, investigation, writing – original draft. Sindy Hu, Chun‐Yu Cheng, Shyue‐Luen Chang, Chau‐Yee Ng, Ya‐Ching Chang, Min‐Hui Chi, Jennifer Wu, Sze‐Wen Ting, Fang‐Ying Wang: data curation, investigation. Yau‐Li Huang: writing – review and editing, supervision, project administration.

## Funding

The authors have nothing to report.

## Ethics Statement

The study protocol was approved by the Institutional Review Board (IRB) of the Chang Gung Medical Foundation (IRB No. 202502152B0). All procedures performed in studies involving human participants were in accordance with the ethical standards of the institutional and/or national research committee and with the 1964 Helsinki declaration and its later amendments or comparable ethical standards.

## Conflicts of Interest

The authors declare no conflicts of interest.

## Data Availability

The data that support the findings of this study are available from the corresponding author upon reasonable request.
